# Transesophageal echocardiogram contributes to high-quality cardiopulmonary resuscitation: a case report

**DOI:** 10.1186/s12871-025-03021-1

**Published:** 2025-03-31

**Authors:** Qiong Wang, Bin Lu

**Affiliations:** Department of Anesthesiology, Zigong Fourth People’s Hospital, 19# Tanmulin Street, Zigong, 643000 Sichuan China

**Keywords:** Perioperative cardiac arrest, Cardiopulmonary resuscitation, Transesophageal echocardiography

## Abstract

**Background:**

Difficulties to identify the cause of cardiac arrest in a short period of time lead to prolonging the time for cardiopulmonary resuscitation (CPR) and to poor survival. Transesophageal echocardiogram (TEE) can assist CPR of long duration and improve outcome.

**Case presentation:**

In this case report, a 50-year-old man was scheduled to undergo a endoscopic cervical discectomy under general anesthesia. The patient suffered a sudden cardiac arrest during the operation, and a high-quality CPR was performed with the the help of TEE. Although the exact etiology of cardiac arrest remained unclear and the CPR was performed for up to 90 min, the patient returned to spontaneous circulation, and was discharged after a month of treatment and rehabilitation, resuming his daily activities. After a one year of follow-up, he still was without any sequelae.

**Conclusions:**

Perioperative cardiac arrest is unpredictable and catastrophic, so high-quality CPR is essential. TEE’s excellent features make it ideal for use on resuscitation and can improve the outcome of cardiac arrest.

**Supplementary Information:**

The online version contains supplementary material available at 10.1186/s12871-025-03021-1.

## Background

The causes of perioperative cardiac arrest may not only be related to the patient’s own disease, but also to anesthesia and surgery [1, 2]. Sometimes it is difficult to identify the cause of cardiac arrest in a short period of time, which may prolong the time for cardiopulmonary resuscitation (CPR) and lead to poor survival [3]. It is crucial to know how to perform a high-quality CPR when the reason is unclear, as it affects the outcome of the patient and whether there will be any sequelae [4]. In this case report, we present a patient who not only successfully returned to spontaneous circulation (ROSC) after a long duration of CPR, but also had a good prognosis with the help of transesophageal echocardiogram (TEE).

## Case presentation

**Fig. 1:**
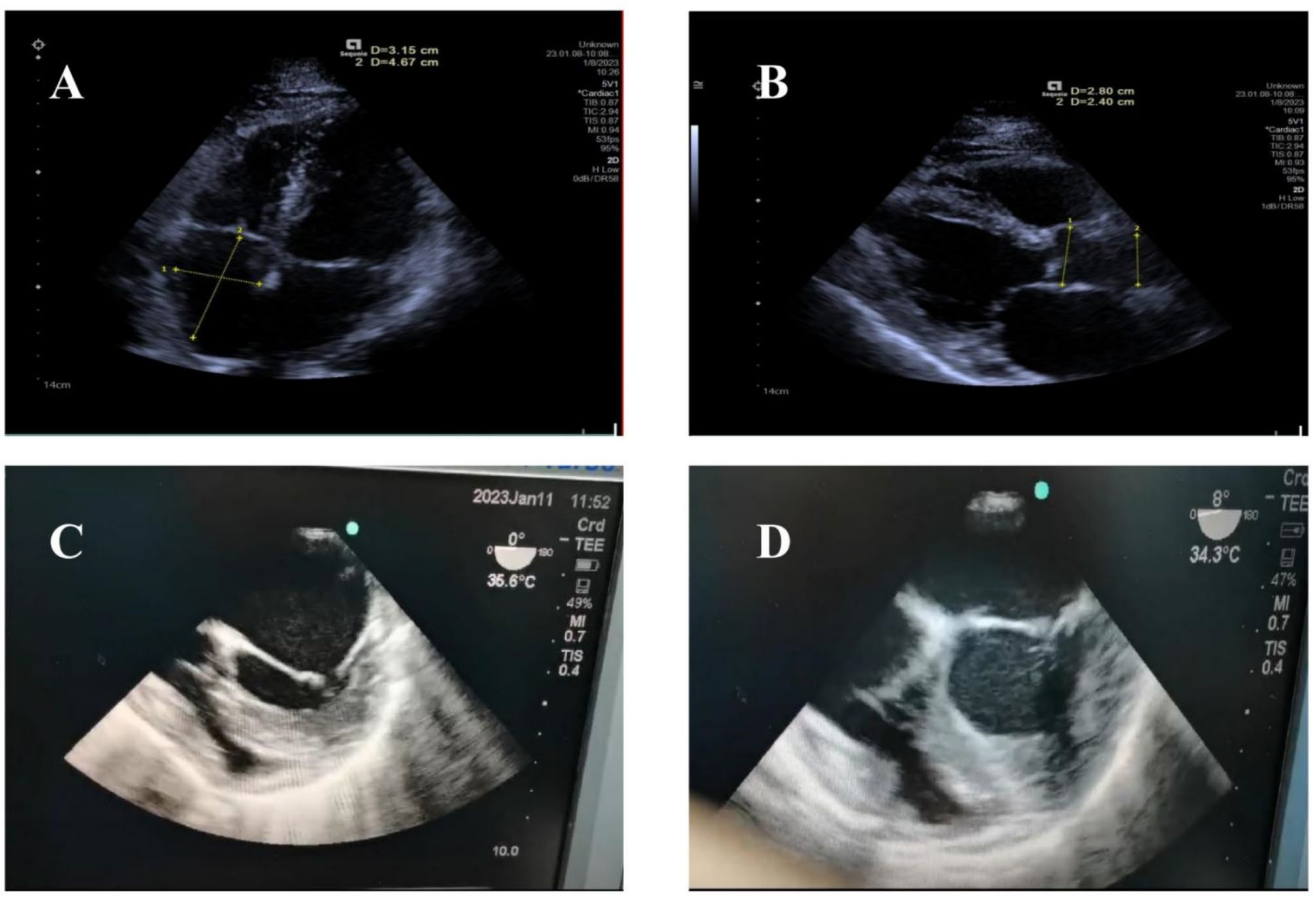
**A**. Preoperative examination; **B**. Preoperative examination; **C**. Isolated valve movement; **D**. Heartbeat recovery

This patient was a 50-year-old man (height: 170 cm; weight: 60 kg) with a cervical radiculopathy in C4 ~ C5. He was suffering from pain and numbness in his left upper extremity, and was scheduled to undergo a cervical discectomy under the spinal endoscopy. The patient was usually in good health and his medical history, physical examination results, and blood chemistry findings were unremarkable. Cardiac ultrasound and lower limb color Doppler ultrasound did not show any abnormalities (Fig. 1A and B). He was considered to be ASA II.

After the patient entered the operating room, a radial artery catheterization was performed to monitor invasive blood pressure (IBP). The patient’s HR was 61 bpm, IBP was 120/70 mmHg, and SpO_2_ was 98% then. Preoperative blood gas analysis was also normal (Supplementary material 3). Anesthesia induction and endotracheal intubation were completed with midazolam 2 mg, propofol 80 mg, etomidate 8 mg, sufentanil 25 ug, and rocuronium 50 mg. Then the patient was turned to prone position and prepared for disinfection. During this period, 1 g of aminotoluene acid was given intravenously. During the operation, 2% sevoflurane was inhaled to maintain anesthesia, and rocuronium 10 mg and sufentanil 5 ug were added every hour.

The patient’s vital signs at the beginning of the surgery were as follows: IBP 100/65 mmHg, HR 60 bpm, and SpO_2_ 100%. Approximately 30 min later, his HR gradually increased to 85 bpm, then increased again to 97 bpm after 1 min, and his IBP remained around 100/59 mmHg. Approximately 1 min later, the ECG and SpO_2_ waves suddenly disappeared while IBP was 30/24 mmHg. His heartbeat was absent on palpation of the right brachial artery, and the surgeon immediately began to perform chest compressions. Then he was turned to the supine position after the surgical area was covered, and 1 mg epinephrine was administered intravenously every 5 min [5]. The patient had a physical examination and an esophageal ultrasound probe was placed to find out possible causes. No obvious abnormalities were found in the pericardium and four cardiac chambers under ultrasound. CPR was performed without any interruption while in the meantime the exact etiology of cardiac arrest remained unclear. We adjusted the amplitude and position of chest compressions so that the mean arterial pressure (MAP) reached to 60 mmHg. Isolated valve movement was the first to appear after 90 min of CPR (Fig. 1C). A few minutes later, synchronous movement of the ventricular wall occurred, and the ECG showed electrical activity. Slowly, the heart began to resume (Fig. 1D), and chest compressions were stopped. The patient then was transferred intubated and ventilated to the ICU for further supportive treatment.

That evening, the patient could open his eyes when his name was called. On day 3, the patient could nod his head to respond. On day 10, his tracheal tube was removed and he could recall events prior to the operation. On day 32, the patient’s renal function had recovered well and he no longer required dialysis. After ensuring that his condition was stable, the patient was discharged.

## Discussion

A healthy middle-aged man suffered a cardiac arrest and a prolonged CPR during cervical surgery. The patient required 32 days to recover from renal failure, and eventually had no sequelae. Mortality increases with the duration of CPR [6] but a good prognosis is often due to the young age of patients, good functional status, and reversible causes of cardiac arrest, and immediate high-quality CPR.

Cardiac arrest in the operating room is typically due to factors that include hypovolemia, drugs, electrolyte disturbance, hypoxia, surgical operation, allergy, and heart disease [7]. According to the examination of TEE, evidence of pulmonary embolism was insufficient because no signs of right ventricular enlargement and dysfunction were found (Supplementary material 1, 2). Although no skin manifestations and airway problems were observed, fatal anaphylaxis cannot be ruled out because of the lack of testing of samples, such as tryptase. Possible culprits could be aminotoluene acid and rocuronium, which are frequently considered to be the main allergens during the perioperative period [8]. The exact cause of the patient’s cardiac arrest still remains to be elucidated.

This difficult but ultimately successful administration of CPR was due primarily to the use of TEE. Compared to transthoracic echocardiography (TTE) and manual pulse checks, TEE ensures continuity of the CPR process by shortening the interruptions for chest compressions [9]. In addition, TEE provides the medical team with the best location for chest compressions. The left ventricle (LV) compressions have been found to improve hemodynamics and increase the rate of ROSC compared to aortic root chest compressions [10]. The left ventricular outflow tract (LVOT) and aortic valve must be open during the compression phase [11, 12]. However, the LV is not always placed below the sternal center where in 50-80% aortic root, aortic valve, and the LVOT can also be located [13]. Another advantage of TEE is the ability to make bedside diagnosis without interrupting CPR. Reliable TEE imaging can assist in ruling out cardiovascular causes, including cardiac tamponade, intracardiac thrombosis, pulmonary embolism, and fine ventricular fibrillation, and assist in resolving risk factors in a timely manner [14–16]. In addition, TEE can provide real-time feedback on the quality of CPR, and guide the CPR process [17, 18]. During this CPR, isolated valve movement was first observed, followed by electrical activity and weak myocardial contraction. Without the benefit of TEE’s real-time monitoring, the information received lags behind, which may affect treatment decisions and prognosis. Overall, TEE monitoring can provide continuous myocardial activity images, identify most reversible causes of cardiac arrest, shorten the interruption of CPR, optimize the quality of chest compressions, and guide the resuscitation process [19].

Compared to NIBP, IBP is timely and synchronous for the judgment of cardiac arrest, which can help us to initiate CPR as early as possible. Furthermore, IBP plays an important role in personalized hemodynamic-directed CPR. It achieves the predetermined hemodynamic goals, including systolic blood pressure, diastolic blood pressure, and coronary perfusion pressure by indicating the chest compression depth and vasopressor dose [20]. Based upon the prognosis of the patient, he received adequate cerebral perfusion and coronary perfusion during the long duration of CPR.

As shown in this case report, the use of TEE in cardiac arrest should be part of resuscitation guidelines to assist management of perioperative CPR. It provides the medical team with bedside diagnosis and high-quality CPR, and serves as the basis for clinical management and decision-making in the perioperative period. Future studies are needed to compare the difference in resuscitation time between CPR with and without TEE in order to discuss the importance of TEE in terms of outcomes.

## Electronic supplementary material

Below is the link to the electronic supplementary material.

Supplementary Material 1

Supplementary Material 2

Supplementary Material 3

Supplementary Material 4

## Data Availability

The datasets are available from the corresponding author on request. All data is provided within the manuscript and supplementary information files.

## Abbreviations

cm: Centimetre
kg: Kilogram
µg: Microgram
mg: Milligram
NIBP: Noninvasive blood pressure
HR: Heart rate
bpm: Beats per min
ECG: Ectrocardiogram
SpO_2_: Saturation of peripheral oxygen
ICU: Intensive care unit
ASA: American society of anesthesiologists physical status classification
