# Supplementary material for: Transesophageal echocardiogram contributes to high-quality cardiopulmonary resuscitation: a case report
Source: BMC Anesthesiol. 2025 Mar 31;25:143. doi: 10.1186/s12871-025-03021-1 (PMC11956450; doi:10.1186/s12871-025-03021-1)
Supplement: Supplementary file 3 — Supplementary Material 3 [file 12871_2025_3021_MOESM3_ESM.docx]

|  | Before surgery | During CPR | ROSC |
| --- | --- | --- | --- |
| pH | 7.355 | 7.59 | 7.29 |
| pCO2 | 44.6 | 32.6 | 34.0 |
| pO2 | 71.8 | 102.1 | 104.4 |
| HCO3- | 24.3 | 31 | 16.3 |
| BE(B) | -1.4 | 9.4 | -9.0 |
| Hct | 42 | 46 | 50 |
| Hb | 144 | 155 | 170 |
| Na+ | 139.6 | 146.1 | 142.7 |
| K+ | 4.31 | 4.89 | 4.21 |
| Ca++ | 1.21 | 1.26 | 1.15 |
| Cl- | 107 | 105 | 106 |
| Glu | 5.6 | 16.4 | 20.6 |
| Lac | 0.95 | 9.01 | 14.19 |

1. Preoperative blood gas analysis was obtained with the patient inhaling air.
2. Blood gas analysis during CPR was measured after the infusion of sodium bicarbonate solution.
3. ROSC: Return to spontaneous circulation.
